# The Physical and Psychological Effects of Telerehabilitation-Based Exercise for Patients With Nonspecific Low Back Pain: Prospective Randomized Controlled Trial

**DOI:** 10.2196/56580

**Published:** 2024-09-06

**Authors:** Weihong Shi, Yuhang Zhang, Yanyan Bian, Lixia Chen, Wangshu Yuan, Houqiang Zhang, Qiyang Feng, Huiling Zhang, Diana Liu, Ye Lin

**Affiliations:** 1 Department of Rehabilitation Medicine, Peking Union Medical College Hospital, Chinese Academy of Medical Sciences and Peking Union Medical College, Beijing, China; 2 Department of Orthopedics, Peking Union Medical College Hospital, Chinese Academy of Medical Sciences and Peking Union Medical College, Beijing, China; 3 Jiakang Zhongzhi Technology Company, Beijing, China; 4 University of Chicago, Chicago, IL, United States

**Keywords:** nonspecific low back pain, telerehabilitation, physical therapy, low back pain, back pain, psychological, exercise, randomized controlled trial, efficacy, medical infrastructure, pain intensity, quality of life, health survey, therapeutic, mobile phone

## Abstract

**Background:**

Physical therapy has demonstrated efficacy in managing nonspecific low back pain (NLBP) among patients. Nevertheless, the prevalence of NLBP poses a challenge, as the existing medical infrastructure may be insufficient to care for the large patient population, particularly in geographically remote regions. Telerehabilitation emerges as a promising method to address this concern by offering a method to deliver superior medical care to a greater number of patients with NLBP.

**Objective:**

The purpose of this study is to demonstrate the physical and psychological effectiveness of a user-centered telerehabilitation program, consisting of a smartphone app and integrated sensors, for patients with NLBP.

**Methods:**

This was a single-center, prospective, randomized controlled trial for individuals with NLBP for a duration exceeding 3 months. All participants were assigned randomly to either the telerehabilitation-based exercise group (TBEG) or the outpatient-based exercise group (OBEG). All participants completed a 30-minute regimen of strength and stretching exercises 3 times per week, for a total of 8 weeks, and were required to complete assessment questionnaires at 0, 2, 4, and 8 weeks. The TBEG completed home-based exercises and questionnaires using a telerehabilitation program, while the OBEG completed them in outpatient rehabilitation. The Oswestry Disability Index (ODI) served as the primary outcome measure, assessing physical disability. Secondary outcomes included the Numeric Pain Rating Scale, Fear-Avoidance Beliefs Questionnaire, and 36-item Short-Form Health Survey.

**Results:**

In total, 54 of 129 eligible patients were enrolled and randomly assigned to the study. The completion of all the interventions and assessments in the TBEG and OBEG was 89% (24/27) and 81% (22/27). The findings indicate that no statistical significance was found in the difference of ODI scores between the TBEG and the OBEG at 2 weeks (mean difference −0.91; odds ratio [OR] 0.78, 95% CI −5.96 to 4.14; *P*=.72), 4 weeks (mean difference −3.80; OR 1.33, 95% CI −9.86 to −2.25; *P*=.21), and 8 weeks (mean difference −3.24; OR 0.92, 95% CI −8.65 to 2.17; *P*=.24). The improvement of the ODI in the TBEG (mean −16.42, SD 7.30) and OBEG (mean −13.18, SD 8.48) was higher than 10 after an 8-week intervention. No statistically significant differences were observed between the 2 groups at the 8-week mark regarding the Fear-Avoidance Beliefs Questionnaire (mean difference 8.88; OR 1.04, 95% CI −2.29 to 20.06; *P*=.12) and Numeric Pain Rating Scale (mean difference −0.39; OR 0.44, 95% CI −2.10 to 1.31; *P*=.64). In the subgroup analysis, there was no statistically significant difference in outcomes between the 2 groups.

**Conclusions:**

Telerehabilitation interventions demonstrate comparable therapeutic efficacy for individuals with NLBP when compared to conventional outpatient-based physical therapy, yielding comparable outcomes in pain reduction and improvement in functional limitations.

## Introduction

### Background

Nonspecific low back pain (NLBP) is a broad category of low back pain (LBP) lacking identifiable etiology [1]. The Global Burden of Disease study report indicates that by 2020, the global prevalence of LBP will exceed 619 million, representing a 60% increase since 1990, with projections reaching 843 million by 2050 [2]. Years lived with disability is an index measuring the average duration of life lived with disability due to a disease from onset to death [3]. The Global Burden of Disease study reveals that LBP ranks first among 291 diseases in terms of age-standardized years lived with disability [4]. By 2020, 69 million individuals will experience limited life expectancy due to disability caused by LBP [2].

It is estimated that the annual economic burden for physical therapy of patients with LBP exceeded US $2.41 billion [5]. Nonspecific chronic LBP includes individuals experiencing pain exceeding 3 months, constituting 85% of the overall population with NLBP [6]. Therefore, it is urgent and necessary to explore innovative treatment modalities for individuals with NLBP.

Numerous clinical guidelines recommend incorporating exercise routines into the treatment of individuals with NLBP. This recommendation is based on the substantial pain relief and improved physical function observed in patients with NLBP through exercise interventions. These interventions also tend to have fewer adverse effects compared to pharmaceutical and surgical approaches [7-10].

Under the traditional clinic-based exercise model, patients are required to participate consistently in structured exercise programs. These programs are supervised by qualified physical therapists (PTs) over an extended period. However, the traditional clinic-based exercise model faces challenges because a substantial proportion of patients with NLBP face barriers to completing a structured exercise program due to time constraints, transportation limitations, and geographical challenges; in addition, a large proportion of individuals with NLBP, who live in remote areas, have no access to qualified exercise guidance because the medical resource is undistributed, especially in resource-limited countries [11-13].

As a result, the traditional clinic-based exercise model encounters difficulties in addressing the diverse needs of this patient population [14,15]. Given these circumstances, the telerehabilitation-based exercise model, incorporation of home-based exercises into a telerehabilitation program, emerges as a promising and effective strategy to address the previously mentioned challenges associated with managing NLBP. The telerehabilitation-based exercise model provides patients with a digitalized exercise plan, enabling patients with NLBP to complete their home exercise regimen promptly. In this model, patients’ exercise performance is recorded, and any issues encountered are promptly addressed by professional health care providers. Compared to traditional clinic-based exercise models, the telerehabilitation-based model saves time, money, and medical resources.

In recent years, numerous research teams have studied telerehabilitation strategies for individuals with NLBP [16-21]. In the United States, Shebib et al [18] pioneered a comprehensive digital care program encompassing education, sensor-guided physiotherapy, aerobic exercise, and cognitive behavioral therapy tailored for patients with NLBP. Their investigation revealed superior therapeutic outcomes within the digital care program group compared to the control group [19]. In Germany, Toelle et al [19] developed the Kaia app (Kaia Health Corp) specifically designed for patients with NLBP. Results indicated that individuals receiving exercise guidance through the Kaia app exhibited significant pain relief and improvements in physical function compared to the control group [19].

Similarly, in the United Kingdom, Fatoye et al [20] integrated telerehabilitation with the McKenzie exercise approach. Remarkably, the telerehabilitation group achieved therapeutic outcomes equivalent to outpatient rehabilitation. It also demonstrated a lower average medical cost per patient compared to the outpatient group [20]. In resource-limited nations, such as China, the widespread adoption of telerehabilitation is important because of the large patient population and the lack of physical therapy services.

Furthermore, it is worth noting that psychosocial risk factors have a more significant impact on predicting pain-related outcomes in cases of NLBP compared to biomedical factors [22,23]. One widely accepted conceptual framework for understanding how psychosocial factors influence pain-related outcomes is the Fear-Avoidance Model [24]. According to the Fear-Avoidance Model, anxiety, depression, fear, and catastrophizing are risk factors that contribute to pain-related disability [25,26]. Marshall et al [27] found that a significant number of patients with NLBP did not experience improvements in pain intensity or limb disability after receiving professional exercise guidance and participating in weekly exercises. These patients were more susceptible to anxiety and fear [28]. In the traditional clinic-based exercise model, health care professionals, such as PTs, assist patients in correctly understanding pain and addressing their concerns to prevent the occurrence of anxiety and fear [28]. However, it remains unclear whether a telerehabilitation-based exercise model can reduce pain-related fear and anxiety in patients with NLBP.

### Objective

The research team used the Healbone Intelligent Rehabilitation System (HIRS), comprised of a smartphone app and integrated sensors. The primary objective is to evaluate the efficacy of this intervention. The program guides and monitors patients with NLBP, as they engage in a structured home-based exercise regimen. This study measures the program’s impact on both the physical and psychological dimensions of NLBP management.

## Methods

### Ethical Considerations

This study has been approved by the ethics committees of the Peking Union Medical College Hospital (I-23PJ151) and registered in the Chinese Clinical Trial Registry (ChiCTR2300068984).

### Trial Design

This study was a single-center, 2-arm, parallel-group, randomized controlled trial (RCT; participant-blinded) with 1:1 RCT, conducted in Peking Union Medical College Hospital, Beijing, China. All patients were assessed on pain, function, quality of life, and fear-belief avoidance. Assessments occurred at 0, 2, 4, and 8 weeks.

### Inclusion and Exclusion Criteria

In this study, all participants were recruited from Peking Union Medical College Hospital. Two physicians selected patients with NLBP who met the inclusion and exclusion criteria and referred them to PTs. These patients were thoroughly informed about the purpose, procedures, and potential risks of the trial. Additionally, patients did not participate in any other medical interventions for LBP other than the exercise intervention of this trial until the study’s completion. Upon obtaining informed consent, patients were included in the study.

Written informed consents were obtained from all patients. The inclusion and exclusion criteria are shown in Textbox 1.

Textbox 1. **Inclusion and exclusion criteria for the study.**

**Inclusion criteria**

- Aged between 18 and 60 years
- Numeric Pain Rating Scale equal to or greater than 3 points
- Oswestry Disability Index equal to or greater than 15 points
- Ongoing pain for at least 3 months
- Able to use a smartphone and complete the exercise protocol independently
- Those who could sign the informed consent independently

**Exclusion criteria**

- Patients with spinal deformity, spinal structure slip, spinal fracture history, and spinal tumor
- Diagnosed with rheumatoid arthritis and ankylosing spondylitis
- Patients with herniated disk
- Pregnancy
- Patients who receive other treatments before the experiments, including nonsteroid anti-inflammatory drugs or plasters, physical agents therapy, and acupuncture

### Sample Size Calculation

The sample size was calculated using PASS 11 (NCSS Corp). Based on the principle of noninferiority RCTs [29] and previous clinical studies [20,30], the mean difference in the Oswestry Disability Index (ODI) between the telerehabilitation-based exercise group (TBEG) and the outpatient-based exercise group (OBEG) was 5, the SD was estimated to be 6 for both groups, and the noninferiority margin for the ODI was 10. A sample size of 38 was required based on a bilateral α=.05 and β=.2, and a sample size of 54 was required to account for a 30% dropout rate.

### Blinding and Randomization

In total, 54 participants were assigned in a randomized manner, with equal distribution, to either the TBEG or the OBEG through a platform for randomization. Subsequently, based on the results derived from the platform (eg, C, T, C, T, T, and C), slips of paper labeled with the letters “T” and “C” were placed into sealed, opaque, and identically sized envelopes. After completing the baseline measurements for all participants, the envelopes were sequentially opened to reveal the group assignments. The allocation sequence was prepared by 2 researchers with no involvement in the study using a blocked randomization model.

### Intervention

#### Overview

The exercise plan for patients with NLBP in both TBEG and OBEG was identical. Both consist of muscle strengthening and stretching exercises to increase lumbar stability, coordination, and posture keeping. The detailed exercise plan is shown in Multimedia Appendix 1. Before the initiation of this trial, 2 PTs were trained in three 40-minute sessions.

#### Telerehabilitation-Based Exercise Group

**Figure 1.**
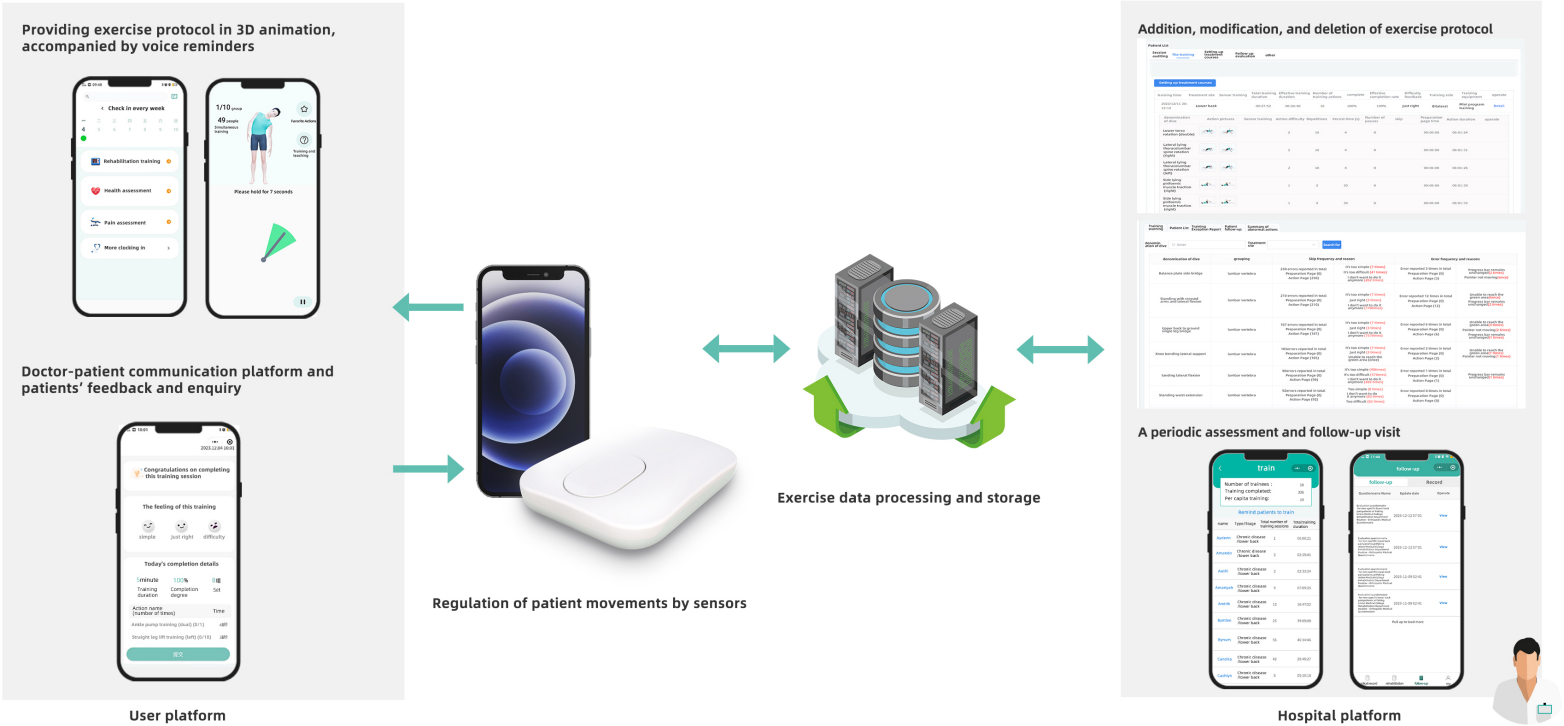
The 3 different parts of the Healbone Intelligent Rehabilitation System. The doctor’s portal could be used to create and modify exercises, monitor training progress, and view patient data. The patients could use the user’s portal to complete the prescribed exercises, view educational materials, and provide feedback to the physical therapists. Finally, the transmitter portal encrypts and transmits the data collected, ensuring the overall system’s integrity.

The HIRS was designed based on a user-centered theory to provide patients with a platform for self-management interventions. Additionally, the HIRS system is made available at no cost to all participants in this study. Figure 1 illustrates the 3 distinct components of HIRS: the physician portal, the user portal, and the transmission portal.

Prior to the commencement of the experiment, professional medical personnel created specific videos for each training exercise in the rehabilitation program and uploaded them to the HIRS along with detailed instructions. At the onset of the experiment, an app was installed on the smartphones of the patients in the TBEG, through which they registered personal accounts. Subsequently, during the initial session, PTs sent digital exercise training protocols to the patients’ personal accounts and educated them on the correct use of the app and sensors for home-based exercises.

Each time the patients engaged in the exercises, they were required to access the app via their personal accounts and calibrate the sensors to accurately perform each exercise within the regimen. Upon initiation of the exercise, the patients were to follow the instructions provided in the video to complete each action in the regimen. If a patient failed to exercise, the system would automatically send a reminder and notify the PTs, who would then contact the patient to ascertain the reason for nonparticipation. Concurrently, if an individual in the TBEG sought advice from the PTs regarding concerns or inquiries related to back pain, the PTs would provide the patient detailed responses to prevent the individual from experiencing fear or anxiety.

Over an 8-week period, all patients in the TBEG were mandated to complete exercise sessions every other day, 3 times a week, with each session lasting 30 minutes. The HIRS transmission portal collected the results of the patients’ assessments and automatically recorded their exercise performance, including the duration of each session and the frequency of weekly exercises.

Finally, patients were required to complete digital assessment questionnaires via the app at weeks 0, 2, 4, and 8. The validity of HIRS had been verified by 25 patients before the trial.

#### Outpatient-Based Exercise Group

In the OBEG, patients underwent a consistent 30-minute exercise regimen every 2 days under the PTs’ supervision, with sessions scheduled thrice weekly. Concurrently, during each hospital visit for exercise guidance, the PTs provide face-to-face consultations to address any questions or concerns the patients may have. Furthermore, assessment questionnaires are administered in the outpatient clinic at baseline (week 0), week 2, week 4, and week 8.

### Outcome Measures

The ODI, as the primary outcome measure, has been verified for reliability and validity [31]. It is commonly used to evaluate physical function. The minimal clinically important difference (MCID) refers to the smallest change in score that patients perceive as beneficial, irrespective of side effects and costs. Bombardier et al [32] determined that the MCID for the ODI score in patients with NLBP is 5. This indicates that an improvement in the ODI score by at least 5 points after the intervention is considered clinically meaningful for the patient.

In addition, a set of secondary outcome measures was also used. These measures include the Numeric Pain Rating Scale (NPRS) for pain evaluation, the 36-item Short-Form Health Survey (SF-36) for quality of life assessment, and the Fear-Avoidance Beliefs Questionnaire (FABQ) to gauge fear-avoidance beliefs related to work and physical activity [33,34]. Previous studies have determined that the MCID for the FABQ in patients with NLBP is 11 [35], and for the NPRS, it is 2 [36]. However, Grönkvist et al [37] established that the MCID for the 8 dimensions of the SF-36 varies among patients with NLBP [37].

The reliability and validity of the Chinese version of the SF-36 and the FABQ have been confirmed [33,34]. The collection of the primary and secondary outcome measures occurred at weeks 0, 2, 4, and 8. Patients in the TBEG completed all assessments through the HIRS, while patients in the OBEG completed these assessments in the outpatient clinic, guided by PTs.

### Statistical Analysis

The outcomes were analyzed following the intention-to-treat approach, and all participants were analyzed according to the original group assignment. Missing data were handled using multiple imputations by chained equations [38]. Besides, subgroup analysis was conducted following per-protocol analyses in this study.

All the data in this study were analyzed using SPSS (version 23.0; IBM Corp). Demographic data are presented as means (SDs) and numbers (percentages). Descriptive statistics, independent sample 2-tailed *t* tests, and chi-square tests were used to analyze participant characteristics. The normality of distribution for all data was tested by an independent sample *t* test. The results of this study are presented as mean, SD, odds ratio (OR), and 95% CI. The statistical analysis was conducted by a researcher who was blinded and not involved in this study.

## Results

### Study Population and Follow-Up

**Figure 2.**
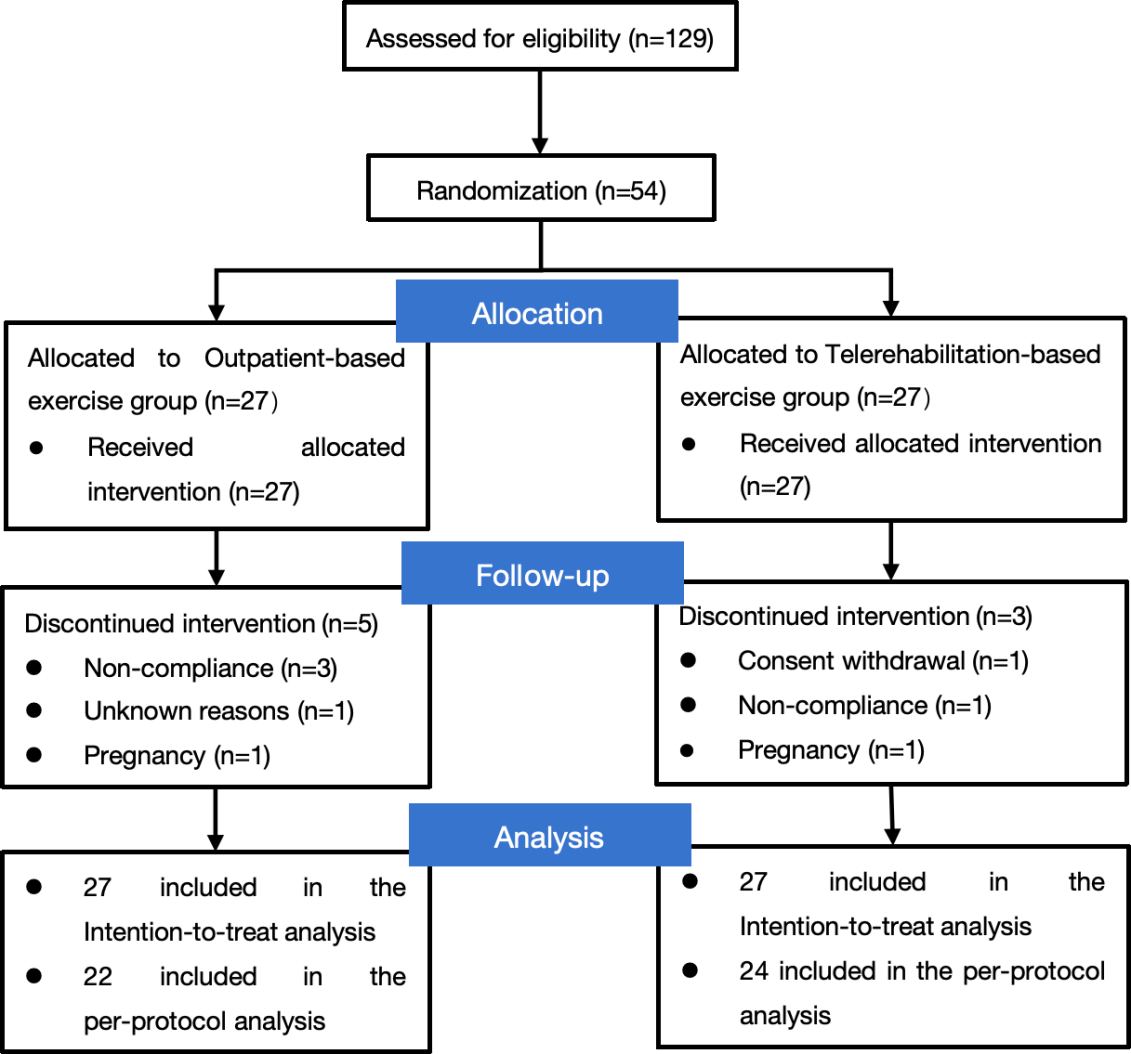
Participant flowchart.

Between March 9, 2023, and November 1, 2023, 129 patients were considered for eligibility. During the initial screening process, 35 patients did not meet the inclusion criteria or met the exclusion criteria. Among the remaining 94 eligible patients, 32 did not consent, and 8 patients withdrew prior to group randomization. Therefore, a total of 54 patients left for the final study. The population was randomly allocated into 2 groups: the TBEG (n=27) and OBEG (n=27), as illustrated in Figure 2.

All patients completed the baseline assessment at week 0 and were asked to complete assessments at weeks 2, 4, and 8. All participants underwent baseline assessments. During the study, 3 patients in the TBEG and 5 patients in the OBEG withdrew due to pregnancy or other reasons. Ultimately, 24 patients in the TBEG and 22 patients in the OBEG completed all treatments and the 8-week follow-up. The completion rates for treatment and assessments in the OBEG were as follows: 96% (26/27) at 2 weeks, 93% (25/27) at 4 weeks, and 81% (22/27) at 8 weeks. In comparison, the completion rates in the TBEG were 100% (27/27) at 2 weeks, 96% (26/27) at 4 weeks, and 89% (24/27) at 8 weeks, as illustrated in Figure 2. Between the 2 groups, patients showed similar clinical and demographic characteristics (Textbox 1).

### Primary Outcomes

The baseline ODI scores showed comparable values between the TBEG and the OBEG (Table 1). The mean ODI improvement for the OBEG was −4.70 (SD 9.20) at week 2, −8.40 (SD 10.13) at week 4, and −13.15 (SD 8.48) at week 8. The mean ODI improvement for the TBEG was −5.61 (SD 7.30) at week 2, −11.43 (SD 8.83) at week 4, and −13.70 (SD 7.30) at week 8. After 8 weeks of intervention, both the OBEG and TBEG demonstrated an improvement in ODI scores exceeding 10 points, indicating clinical significance (Table 2).

**Figure 3.**
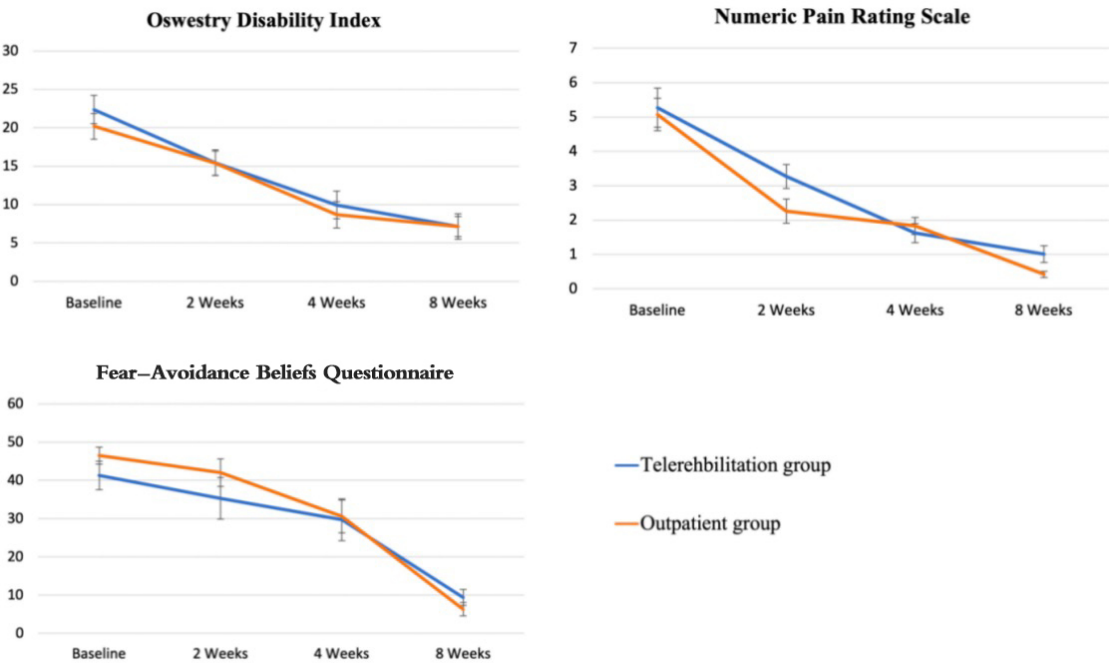
The primary and secondary outcomes Oswestry Disability Index, Numeric Pain Rating Scale, and Fear-Avoidance Beliefs Questionnaire at baseline and 2, 4, and 8 weeks; error bars represent 95% CIs.

At the second week, the difference in ODI score changes between the TBEG and OBEG was 0.41 (OR 0.78, 95% CI −0.58 to 1.39); at the fourth week, the difference was −3.80 (OR 1.33, 95% CI −9.86 to 2.25); and at the eighth week, the difference was −3.24 (OR 0.92, 95% CI −8.65 to 2.17; Table 2). Statistical analysis revealed no significant differences between the TBEG and OBEG at weeks 0, 2, 4, and 8 (Figure 3). Following the 8-week intervention, the improvement in ODI scores in the TBEG was noninferior to that in the OBEG.

**Table 1.** Demographics and baseline characteristics of all participants.

Characteristics		OBEG^a^ (n=27)	TBEG^b^ (n=27)	*P* value
Age (years), mean (SD)		38.23 (11.55)	39.11 (10.45)	.77
Height (m), mean (SD)		1.69 (1.07)	1.65 (0.08)	.08
Weight (kg), mean (SD)		63.96 (9.89)	61.85 (10.50)	.46
BMI (kg/m^2^), mean (SD)		22.39 (3.19)	22.57 (2.98)	.83
**Sex, n (%)**				.41
	Male	12 (57)	9 (43)	
	Female	14 (44)	18 (56)	
Sedentary time per day, mean (SD)		6.69 (2.35)	7.08 (2.73)	.59
Pain duration (months), mean (SD)		10.23 (3.57)	10.11 (3.56)	.90
ODI^c^, mean (SD)		18.80 (6.57)	20.86 (11.40)	.21
NPRS^d^, mean (SD)		5.02 (1.74)	5.42 (3.82)	.27
FABQ^e^, mean (SD)		46.47 (14.36)	41.32 (10.60)	.23
**SF-36** ^**f**^ **, mean (SD)**				
	Physical functioning	61.85 (12.57)	60.96 (2.54)	.87
	Role-physical	45.37 (6.71)	46.15 (7.88)	.94
	Bodily pain	58.52 (17.26)	55.00 (20.45)	.50
	General health	49.63 (16.35)	46.92 (13.12)	.51
	Vitality	73.70 (12.76)	72.50 (15.44)	.76
	Social functioning	80.96 (16.20)	78.08 (18.45)	.55
	Role-emotional	69.33 (2.25)	56.81 (2.83)	.28
	Mental health	66.30 (15.54)	72.73 (15.87)	.14

a OBEG: outpatient-based exercise group.

b TBEG: telerehabilitation-based exercise group.

c ODI: Oswestry Disability Index.

d NPRS: Numeric Pain Rating Scale.

e FABQ: Fear-Avoidance Beliefs Questionnaire.

f SF-36: 36-item Short-Form Health Survey.

**Table 2.** Primary and secondary outcomes for the OBEG^a^ and the TBEG^b^.

		Two weeks					Four weeks					Eight weeks				
		OBEG (n=27), mean (SD)	TBEG (n=27), mean (SD)	Difference of change between 2 groups		*P* value	OBEG (n=27), mean (SD)	TBEG (n=27), mean (SD)	Difference of change between 2 groups		*P* value	OBEG (n=27), mean (SD)	TBEG (n=27), mean (SD)	Difference of change between 2 groups		*P* value
				Mean	OR^c^ (95% CI)				Mean	OR (95% CI)				Mean	OR (95% CI)	
**Primary outcome**																
	ODI^d^	−4.70(9.20)	−5.61(7.30)	−0.91	0.78(−5.96 to 4.14)	.72	−9.93(10.13)	−13.73(8.83)	−3.80	1.33(−9.86 to −2.25)	.21	−13.18(8.48)	−16.42(7.30)	−3.24	0.92 (−8.65 to 2.17)	.24
**Secondary outcomes**																
	NPRS^e^	−2.41(1.95)	−2.00(1.60)	0.41	0.69 (−0.58 to 1.39)	.41	−3.44(2.38)	−3.65(3.77)	−0.21	2.00 (−1.94 to 1.52)	.81	−4.26(3.90)	−4.65(2.01)	−0.39	0.44 (−2.10 to 1.31)	.64
	FABQ^f^	−4.42(17.51)	−6.00(16.21)	−1.58	1.00(−11.03 to 7.88)	.74	−15.91(17.87)	−11.60(16.98)	4.31	0.19(−7.60 to 16.22)	.47	−40.15(13.38)	−31.92(15.07)	8.88	1.04 (−2.29 to 20.06)	.12
**SF-36** ^g^																
	Physical functioning	10.50(16.75)	13.64(23.31)	3.14	1.75 (−9.51 to 15.78)	.62	13.26(15.99)	12.20(24.72)	−1.06	1.38 (−13.05 to 10.92)	.59	16.35(16.62)	15.38(27.81)	−0.96	2.58 (−13.60 to 11.67)	.88
	Role-physical	5.00(32.87)	13.86(41.89)	8.86	1.26(−14.52 to 32.25)	.45	19.06(34.12)	13.64(40.21)	−6.62	1.20(−28.57 to 15.34)	.55	19.69(38.96)	17.27(47.50)	−0.96	1.41 (−25.30 to 23.38)	.94
	Bodily pain	14.71(21.47)	12.27(20.45)	−1.73	1.02 (−14.61 to 11.15)	.79	16.30(21.54)	19.00(26.03)	2.70	0.88 (−12.01 to 17.40)	.71	25.00(15.48)	20.77(26.84)	−4.23	2.03 (−16.84 to 8.38)	.50
	General health	9.50(11.95)	9.32(14.33)	−0.18	1.71(−8.24 to 7.88)	.96	13.26(16.04)	12.80(14.77)	−0.46	1.10(−9.22 to 8.30)	.92	12.88(17.06)	12.50(11.62)	−0.38	1.00(−8.69 to 7.92)	.93
	Vitality	7.40(15.50)	4.32(15.83)	−3.08	1.06(−12.81 to 6.64)	.53	11.30(14.71)	6.00(15.88)	−5.30	1.52(−15.70 to 5.10)	.31	13.75(11.23)	10.00(12.97)	−1.54	1.26(−9.26 to 6.18)	.69
	Social functioning	3.94(22.00)	9.59(19.30)	5.66	1.23 (−7.11 to 18.42)	.38	9.39(22.21)	10.80(16.06)	1.41	0.27 (−10.04 to 12.85)	.81	14.09(17.60)	9.39(21.81)	−4.70	1.44 (−15.37 to 5.98)	.38
	Role-emotional	10.35(14.28)	17.67(24.08)	7.32	0.75 (−17.38 to 32.02)	.55	32.92(38.33)	26.47(18.23)	6.44	0.71 (−20.68 to 33.56)	.64	22.92(15.53)	30.38(29.76)	7.46	0.54 (−17.67 to 32.59)	.55
	Mental health	7.15(16.09)	0.95(15.03)	−6.20	4.80 (−16.41 to 4.02)	.23	23.35(14.41)	6.44(23.88)	−6.91	8.25 (−19.94 to 3.01)	.01	14.46(17.28)	6.50(15.77)	−7.96	4.20 (−17.53 to 1.06)	.10

a OBEG: outpatient-based exercise group.

b TBEG: telerehabilitation-based exercise group.

c OR: odds ratio.

d ODI: Oswestry Disability Index.

e NPRS: Numeric Pain Rating Scale.

f FABQ: Fear-Avoidance Beliefs Questionnaire.

g SF-36: 36-item Short-Form Health Survey.

### Secondary Outcomes

The baseline NPRS scores showed comparable values between the TBEG and the OBEG. Statistical analysis found no significant differences between values for the TBEG and the OBEG at weeks 0, 2, 4, and 8 (Table 1). At week 8, the mean NPRS improvement from baseline was −4.65 (SD 2.01) in OBEG and −4.65 (SD 2.01) in TBEG (Figure 3).

The baseline FABQ scores showed comparable values between the TBEG and the OBEG. Statistical analysis found no significant differences between the values for the TBEG and the OBEG at weeks 0, 2, 4, and 8 (Table 1). At week 8, the mean improvement in FABQ scores from baseline was −40.15 (SD 13.38) in OBEG and −32.48 (SD 15.07) in TBEG (Figure 3).

No statistically significant differences were found in the mean change of the SF-36 scores at weeks 0, 2, 4, and 8 (Tables1  2).

After an 8-week intervention, the NPRS, FABQ, and SF-36 scores in both the TBEG and the OBEG showed significant improvement compared to baseline values. Furthermore, the extent of improvement in NPRS, FABQ, and SF-36 scores in the TBEG was found to be noninferior to that observed in the OBEG.

### Subgroup Analysis

After an 8-week intervention, both the OBEG and the TBEG demonstrated clinically significant improvements in the ODI, with a reduction exceeding 10 points, and no statistical differences were found in the changes of the ODI between 2 groups, demonstrating noninferiority (Table 3).

**Table 3.** Subgroup analysis of primary and secondary outcomes for the OBEG^a^ and the TBEG^b^.

		Two weeks					Four weeks					Eight weeks				
		OBEG (n=26), mean (SD)	TBEG (n=27), mean (SD)	Difference of change between 2 groups		*P* value	OBEG (n=25), mean (SD)	TBEG (n=26), mean (SD)	Difference of change between 2 groups		*P* value	OBEG (n=22), mean (SD)	TBEG (n=24), mean (SD)	Difference of change between 2 groups		*P* value
				Mean	OR^c^ (95% CI)				Mean	OR (95% CI)				Mean	OR (95% CI)	
**Primary outcome**																
	ODI^d^	−5.11(9.26)	−5.61(7.30)	−0.50	0.64 (−5.63 to 4.63)	.84	−9.76(8.54)	−14.16(12.14)	−4.40	0.83 (−10.37 to −2.25)	.15	−12.77(7.04)	−17.48(11.55)	−4.71	1.05 (−10.49 to 1.08)	.11
**Secondary outcomes**																
	NPRS^e^	−2.42(1.98)	−2.00(1.60)	0.42	0.59 (−0.58 to 1.43)	.40	−3.40(2.31)	−3.72(3.84)	−0.32	0.29 (−2.12 to 1.52)	.72	−4.27 (1.78)	−4.87(4.09)	−0.60	0,21 (−2.51 to 1.32)	.53
	FABQ^f^	−4.72(17.81)	−6.00(16.21)	−1.28	0.85 (−10.91 to 8.35)	.79	−15.38(19.94)	−12.54(20.64)	2.84	0.88 (−9.40 to 15.08)	.64	−41.90(24.10)	−30.91(15.27)	10.99	1.57 (−1.17 to 23.16)	.08
**SF-36** ^g^																
	Physical functioning	11.05(16.63)	13.64(23.31)	2.58	0.47 (−10.36 to 15.53)	.69	11.90(14.87)	11.88(24.93)	−0.03	0.94 (−12.60 to 12.54)	.99	14.05(13.29)	16.09(29.62)	2.04	0.79 (−12.16 to 16.24)	.77
	Role-physical	5.79(32.50)	13.86(41.89)	8.07	1.25 (−15.90 to 32.05)	.50	23.57(35.25)	18.13(41.49)	−5.45	1.40 (−28.78 to 17.88)	.64	22.14(38.03)	23.70(49.78)	1.55	1.41 (−25.60 to 28.70)	.91
	Bodily pain	13.68(21.33)	12.27(20.45)	−1.41	1.40 (−14.63 to 11.81)	.83	15.48(21.54)	20.21(27.84)	4.73	0.90 (−10.40 to 19.87)	.53	23.81(16.58)	20.87(29.53)	−2.94	2.03 (−17.70 to 11.82)	.69
	General health	10.00(11.18)	9.32(14.33)	−0.68	1.50 (−8.90 to 7.53)	.87	12.62(14.97)	13.75(5.13)	1.13	0.58(−7.94 to 10.20)	.80	12.38(17.57)	12.83(12.31)	0.45	1.05(−8.87 to 9.76)	.92
	Vitality	8.31(15.50)	4.32(15.83)	−4.00	0.83(−13.82 to 5.83)	.42	11.19(15.48)	5.00(19.78)	−6.19	1.57(−16.98 to 4.60)	.25	12.86(12.41)	13.70(15.54)	0.84	1.54(−7.77 to 9.45)	.85
	Social functioning	4.15(22.)	9.59(19.30)	5.45	1.01(−7.66 to 18.56)	.41	6.57(17.97)	11.25(19.51)	4.67	0.67 (−6.60 to 15.94)	.41	11.42(15.15)	9.16(23.16)	−2.26	0.70(−14.30 to 9.77)	.71
	Role-emotional	10.89(14.69)	17.67(24.08)	6.77	0.78(−18.58 to 32.13)	.59	24.24(43.56)	31.50(50.69)	7.26	0.12(−21.36 to 35.89)	.61	22.00(36.98)	30.00(55.03)	8.00	0.56(−20.81 to 36.81)	.58
	Mental health	7.32(18.19)	0.95(15.03)	−6.36	1.68(−16.85 to 4.13)	.23	23.67(17.28)	15.79(17.38)	−7.88	2.33(−18.32 to 2.57)	.14	15.81(14.18)	8.77(18.96)	−7.04	2.35(−17.39 to 3.31)	.18

a OBEG: outpatient-based exercise group.

b TBEG: telerehabilitation-based exercise group.

c OR: odds ratio.

d ODI: Oswestry Disability Index.

e NPRS: Numeric Pain Rating Scale.

f FABQ: Fear-Avoidance Beliefs Questionnaire.

g SF-36: 36-item Short-Form Health Survey.

## Discussion

### Principal Findings

This study was designed to determine the efficacy of the treatment between the TBEG and the OBEG. After an 8-week intervention, the completion rate was 89% (24/27) in the TBEG and 81% (22/27) in the OBEG. The completion rate of exercise was higher in the TBEG compared to the OBEG. In the primary outcomes, there was no statistically significant difference between the TBEG and the OBEG in improving pain-related physical dysfunction, demonstrating noninferiority of telerehabilitation. However, both groups demonstrated an improvement in the ODI score exceeding 10 points, indicating that both telerehabilitation exercises and outpatient exercises have clinical significance in improving the ODI for patients with NLBP. Regarding secondary outcomes, there were no statistically significant differences in the SF-36, NPRS, and FABQ between the groups; the improvements in the SF-36, NPRS, and FABQ surpassed the MCID. This suggests that both TBEG and OBEG interventions have clinical significance in pain relief, reduction in fear-avoidance beliefs, and enhancement of quality of life following an 8-week intervention with similar efficacy.

### The Efficacy and Benefits of Telerehabilitation for Patients With NLBP

Compared with previous studies [39-41], this study also demonstrates the efficacy of telerehabilitation for patients with NLBP in the improvement of pain intensity, physical disability, and quality of life.

Additionally, we also found that exercise helped patients in both groups to reduce the impact of pain-related fear on work and daily activity after an 8-week intervention. Figure 2, which visually presents the data, indicates significant improvements in both the NPRS and ODI by week 4. Moreover, the FABQ demonstrates a noticeable reduction by week 8, suggesting a delayed improvement in patients’ psychological fear.

Exercise is an important and widely accepted treatment for patients with NLBP [42,43]. To achieve the expected results, it is crucial for patients to consistently follow a prescribed exercise plan for an extended time. However, a study by Palazzo et al [12] found that patients with NLBP face challenges in adhering to home-based exercises. These challenges include factors such as remote locations, difficulties in the exercise program, the patient’s attitude toward exercise, and the lack of supervision and follow-up outside of the hospital. Altogether, these factors reduce the effectiveness of the treatment [11,12].

Hence, we have introduced a telerehabilitation system using a smartphone app combined with sensors. This system is designed to offer better monitoring and follow-up beyond usual care. The exercise plan includes stretching and strength exercises, which have been shown to reduce pain and improve physical function [44-46]. The smartphone app uses visual and audio content to enhance patient experience.

Furthermore, the telerehabilitation system allows patients to receive prompt guidance from PTs. PTs can monitor the real-time physical functional status and exercise progression of patients. The exercise routines span over 8 weeks, with sessions occurring every 2 days, 3 times per week. In contrast to patients in the OBEG who need to schedule appointments with PTs in the clinic, those in the TBEG can complete their exercises at home.

In contrast to the OBEG, patients with NLBP in the TBEG exhibited greater flexibility in their exercise scheduling. Within the scope of this study, they consistently adhered to their exercise regimens in a timely manner, which contributed to improved compliance with exercise plans and benefited patients with NLBP. Moreover, the exercise model based on remote rehabilitation can help individuals save more time and expenses related to hospital visits, offering greater convenience compared to outpatient-based exercise models. Simultaneously, the remote rehabilitation–based exercise model provides patients with NLBP with the opportunity to receive qualified exercise guidance. Finally, this remote rehabilitation–based exercise model alleviates the burden on health care institutions and reduces treatment costs.

The telerehabilitation system enables patients with NLBP to adhere to their treatment plans and allows them to manage their health at home with remote supervision. In long-term follow-ups, Hou et al [47] found that patients using telerehabilitation showed more improvement in functional limitations compared to those relying on the traditional in-clinic method. This is especially promising in areas lacking medical accessibility.

### Limitations

This study was conducted at 1 medical center to compare telerehabilitation with traditional on-site rehabilitation for patients with NLBP. A total of 54 patients participated in the trial, with 27 in the TBEG and 27 in the OBEG. The exercise routines span over 8 weeks, and assessments were scheduled at weeks 0, 2, 4, and 8.

This was a single-center RCT with a relatively small sample size. To address this limitation, the research team plans to conduct subsequent multicenter RCTs in regions with limited medical resources where patients with NLBP have difficulty accessing professional exercise guidance. Additionally, due to the relatively short follow-up period, future studies will involve more participants to investigate the effects of remote rehabilitation–based exercise interventions on patients with NLBP over 6 months, 1 year, or even longer durations [47], focusing on adherence, pain relief, and improvement in pain-related physical dysfunction.

This study demonstrates that remote rehabilitation–based exercise training has therapeutic effects on pain relief and improvement in pain-related physical dysfunction in patients with NLBP. However, there is currently limited research analyzing the factors influencing the efficacy of remote rehabilitation in patients with NLBP, and it remains unclear which types of patients with NLBP are more suitable for remote rehabilitation treatment.

Furthermore, the research team plans to validate the effectiveness of the telerehabilitation program through a multicenter clinical trial. These future efforts are designed to study the benefits of telerehabilitation in managing NLBP, providing valuable insights to the medical community.

### Conclusions

This study confirms that telerehabilitation and traditional outpatient rehabilitation methods produce comparable outcomes for patients with NLBP. Additionally, telerehabilitation reduces time, cost, and medical resources. It exhibits potential as an alternative for patients lacking access to high-quality rehabilitation services.

## Supplementary material

10.2196/56580 Multimedia Appendix 1 Low back pain remote home-based exercise protocol.

10.2196/56580 Checklist 1 CONSORT-eHEALTH checklist (V 1.6.1).

## Abbreviations

FABQ: Fear-Avoidance Beliefs Questionnaire
HIRS: Healbone Intelligent Rehabilitation System
LBP: low back pain
MCID: minimal clinically important difference
NLBP: nonspecific low back pain
NPRS: Numeric Pain Rating Scale
OBEG: outpatient-based exercise group
ODI: Oswestry Disability Index
OR: odds ratio
PT: physical therapist
RCT: randomized controlled trial
SF-36: 36-item Short-Form Health Survey
TBEG: telerehabilitation-based exercise group
