# Supplementary material for: The Physical and Psychological Effects of Telerehabilitation-Based Exercise for Patients With Nonspecific Low Back Pain: Prospective Randomized Controlled Trial
Source: JMIR Mhealth Uhealth. 2024 Sep 6;12:e56580. doi: 10.2196/56580 (PMC11395168; doi:10.2196/56580)
Supplement: Multimedia Appendix 1 [file mhealth-v12-e56580-s001.docx]

Remote Home Exercise Principles

1, All exercises should be completed without any pain, dizziness, or nausea, etc. if a certain exericse causes discomfort or other symptoms, then this exericse should be given up.

2, All exercises need to be completed gently and slowly, avoiding any fast or violent motion!

3, Do not hold breathing during the maintenance phase with all exercises, so as to avoid a sudden increase in blood pressure. Counting out loudly is an efficient method to avoid holding breath.

4, All exercises must be measured, gradually increase the intensity in order to avoid sports injuries!

**The frequency for each exericse: one or two sets of 10-15, one time a day, at least threee times a week as you tolerate.**

**Stretch exercises:**

| **Exercise 1:**  **LOWER TRUNK ROTATIONS** | **LOWER TRUNK ROTATIONS - LTR - WIG WAGS - KNEE ROCKS**  **Lying on your back with your knees bent, gently rotate your spine as you move your knees to the side and then reverse directions and move your knees to the other side. Repeat as you move through a comfortable range of motion.** | 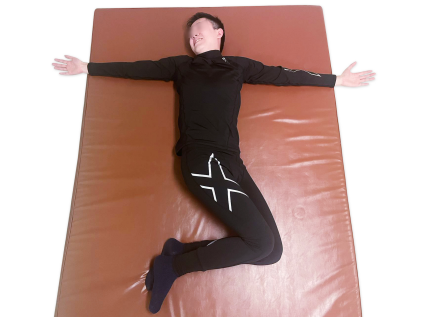 |
| --- | --- | --- |
| **OPEN BOOK** | **OPEN BOOK**  **Lay down on one side with both knees bent up towards the chest at a 90 degree angle. Open up the top arm and reach back until you feel a stretch, forming a "T" shape with your arms. Hold for 3 seconds then close back. Follow your arm with your head as you open and close. Repeat on other side as well.** | 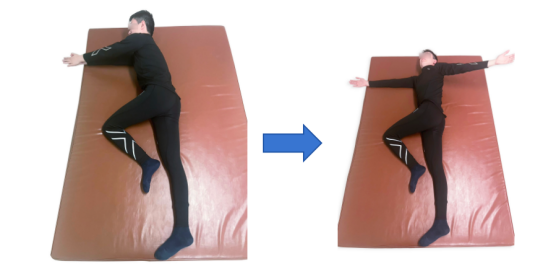 |
| **PIRIFORMIS STRETCH** | **PIRIFORMIS STRETCH**  **While lying on your back with both knees bent, cross your affected leg on the other knee.**  **Next, hold your unaffected thigh and pull it up towards your chest until a stretch is felt in the buttock.**  **Vice versa, 30 seconds hold for each side** | 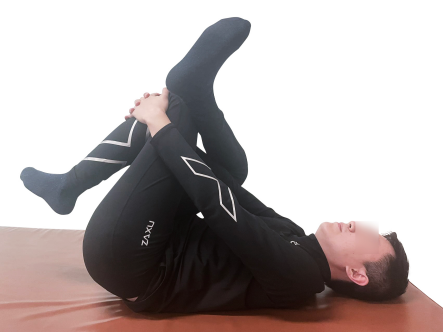 |
| **PRONE ON ELBOWS - POE** | **PRONE ON ELBOWS - POE**  **Lying face down, slowly press up and prop yourself up on your elbows. Hold for 15-30 seconds, lower back down and repeat.** | 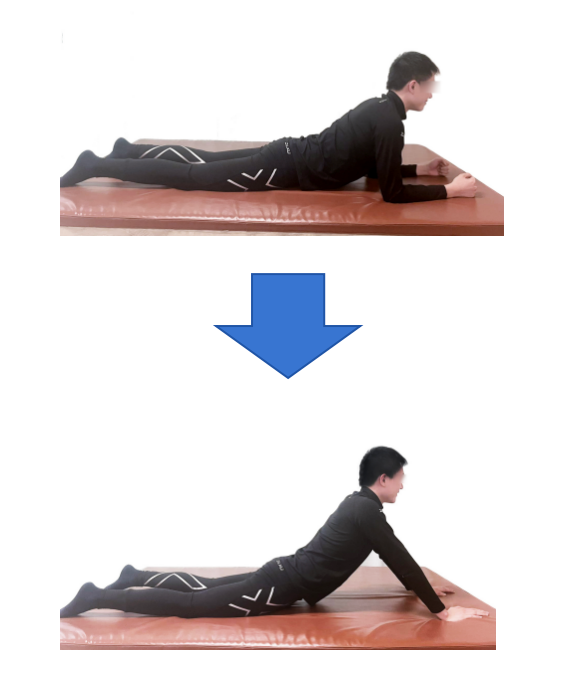 |
| **CAT CAMEL** | **CAT CAMEL**  **While on your hands and knees in a crawl position, raise up your back and arch it towards the ceiling like an angry cat.**  **Next return to a lowered position and arch your back the opposite direction.** | 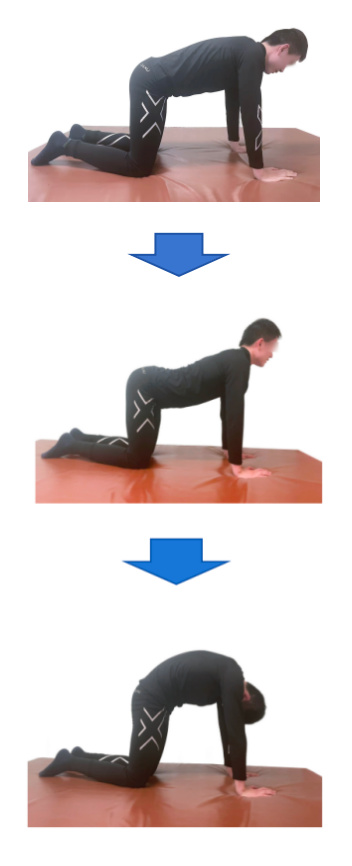 |
| **CHILD POSE - PRAYER STRETCH** | **CHILD POSE - PRAYER STRETCH**  **While in a crawl position, slowly lower your buttocks towards your feet until a stretch is felt along your back and or buttocks.** | 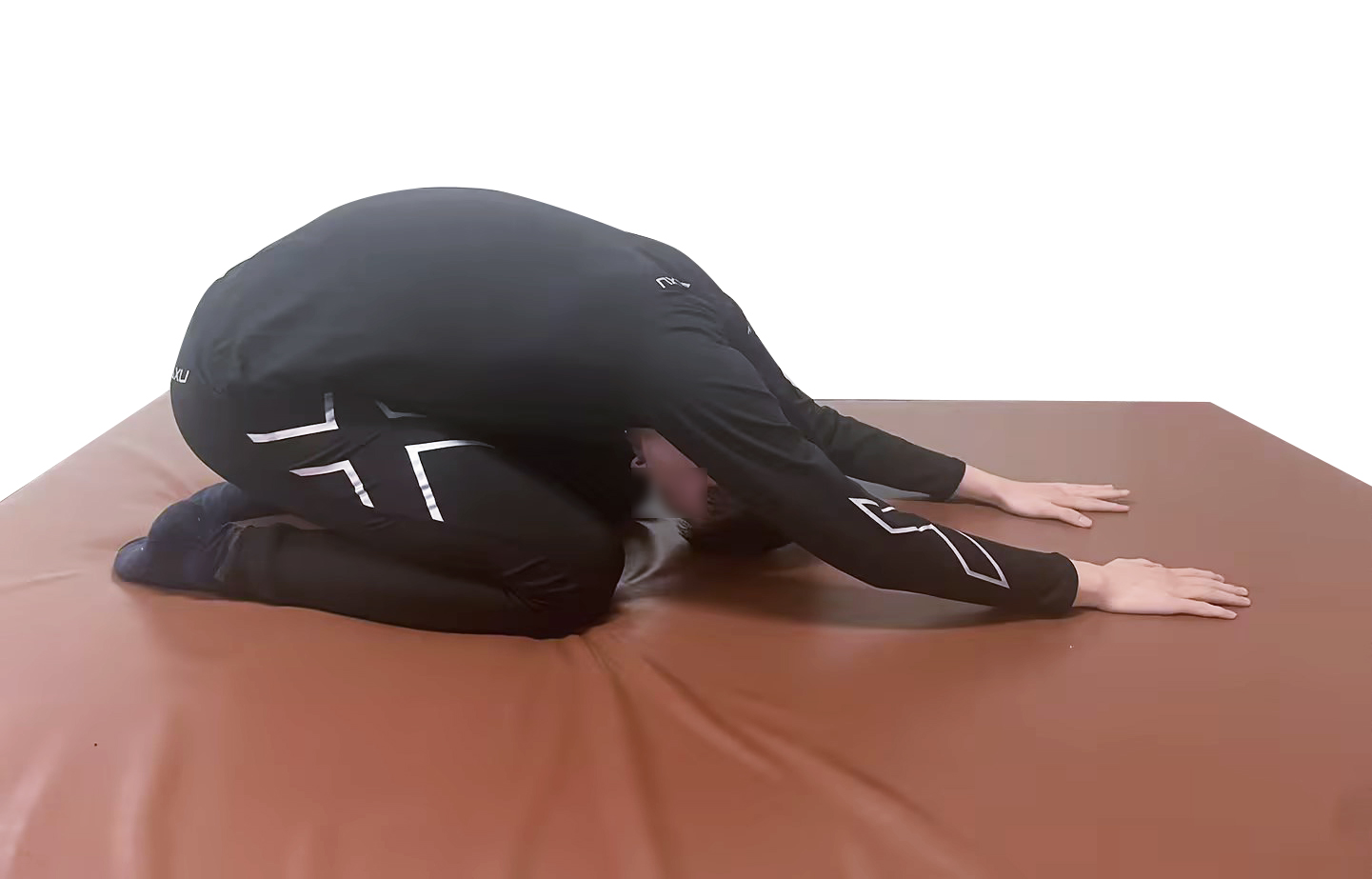 |

Strengthening exercises:

| **BRIDGE - BRIDGING** | **BRIDGE - BRIDGING**  **While lying on your back with knees bent, tighten your lower abdominal muscles, squeeze your buttocks and then raise your buttocks off the floor/bed as creating a "Bridge" with your body. Hold and then lower yourself and repeat.**  **Progress by single leg bridges:**  **SINGLE LEG BRIDGE**  **While lying on your back with your knees bent, extend one knee as shown.**  **Next, raise your buttocks off the floor/bed.**  **Try and maintain your pelvis level the entire time.** | 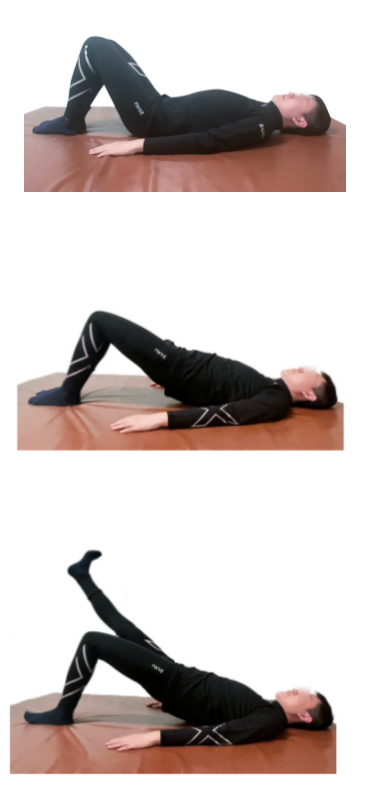 |
| --- | --- | --- |
| **STRAIGHT LEG RAISE - SLR** | **STRAIGHT LEG RAISE - SLR**  **While lying, raise up your leg with a straight knee and a dorsiflexed ankle untile feeling stretch on back of the raised leg. Keep flat back and both knees straight the entire time.** | 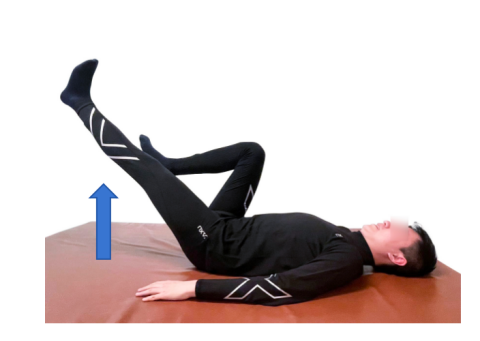 |
| **HIP ABDUCTION - SIDELYING** | **HIP ABDUCTION - SIDELYING**  **While lying on your side, slowly raise up your top leg towards the sky. Keep your knee straight and maintain your toes pointed forward the entire time. Keep your leg in-line with your body.**  **The bottom leg can be bent to stabilize your body.** | 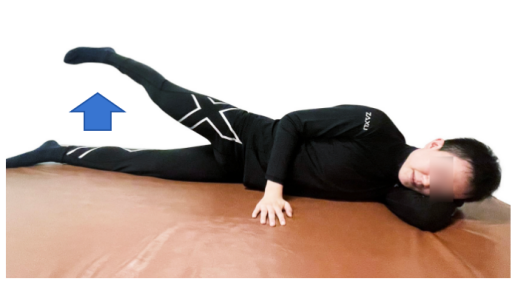 |
| **SUPINE BICYCLE** | **SUPINE BICYCLE - SUPINE HIP EXTENSION**  **Lie on your back with your knees bent. Raise up both feet so your hips are at 90 degrees and your knees are at 90 degrees. Use your stomach muscles to keep your back flat during the exercise. Start by straightening one of your legs out away from you. Then, return the leg back to starting position and repeat on the other side. Repeat.** | 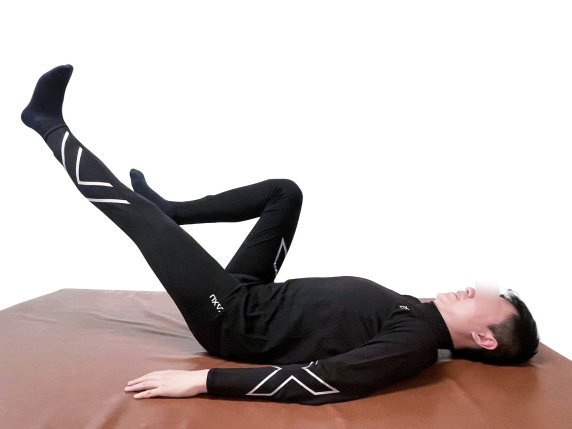 |
| **BIRD DOG** | **BIRD DOG**  **QUADRUPED ALTERNATE ARM AND LEG:**  **While in a crawling position, tighten/brace at your abdominal muscles and then slowly lift a leg and opposite arm upwards. Your hip will move into hip extension on the way up. Lower leg and arm down and then repeat with opposite side.**  **Maintain a level and stable pelvis and spine the entire time.** | 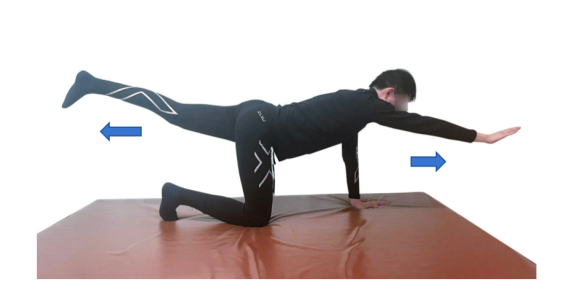 |
| **DEAD BUG** | **DEAD BUG**  **While lying on your back with your knees and hips bent to 90 degrees, use your stomach muscles and maintain pelvic neutral position. Do not allow your spine to move.**  **Hold pelvic neutral and then slowly straighten out a leg without touching the floor. At the same time raise an opposite arm over head. Do not allow your spine to arch during this movement.**  **Return to starting position and then repeat on the opposite side.** | 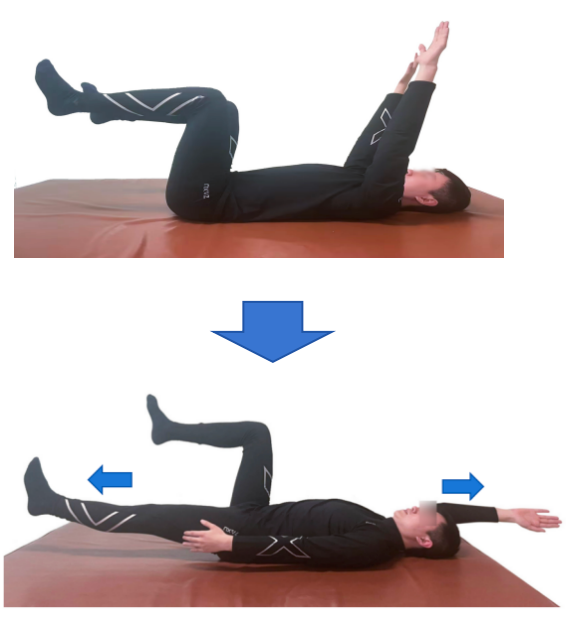 |
| **PLANK** | **PLANK**  **While lying face down, lift your body up on your elbows and toes. Try and maintain a straight spine. Do not allow your hips or pelvis on either side to drop. Maintain pelvic neutral position the entire time.** | 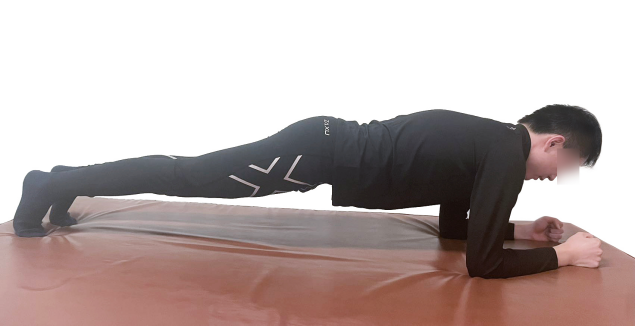 |
| **SIDE PLANK** | **SIDE PLANK**  **Stack your feet with bent knees and place your elbow directly your shoulder. Brace your glutes and core, then lift your torso up off the ground into a straight line as shown. Do not let your pelvis dip down towards the ground. You can place a foam-roller under your hips as added feedback to prevent your body from sagging during this exercise.**  **Try not to rotate through the spine during this exercise and focus on increasing your hold time as tolerated.**  **Progress to sraight knee side plank** | bent knee side plank and straight leg side plank  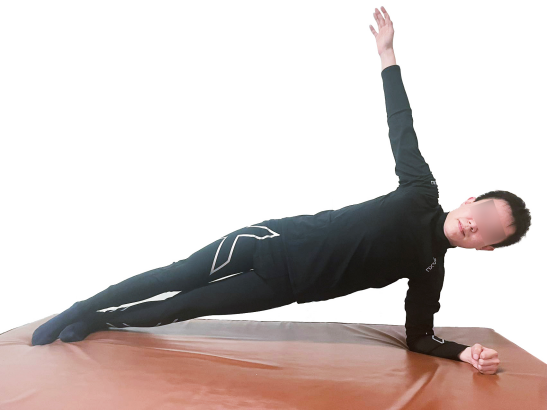 |
