# Supplementary material for: The Physical and Psychological Effects of Telerehabilitation-Based Exercise for Patients With Nonspecific Low Back Pain: Prospective Randomized Controlled Trial
Source: JMIR Mhealth Uhealth. 2024 Sep 6;12:e56580. doi: 10.2196/56580 (PMC11395168; doi:10.2196/56580)
Supplement: Checklist 1 [file mhealth-v12-e56580-s002.pdf]

**CONSORT-EHEALTH Checklist V1.6.2 Report**	**Manuscript Number**	56580

(based on CONSORT-EHEALTH V1.6), available at [http://tinyurl.com/consort-ehealth-v1-6].

Date completed
2/2/2024 2:44:07
by
Weihong SHI

The Physical and Psychological Effects of Telerehabilitation-based Exercise for Patients with Non-specific Low Back Pain: A Prospective Randomized Controlled Trial

TITLE

1a-i) Identify the mode of delivery in the title

"Mobile-based" in the title

1a-ii) Non-web-based components or important co-interventions in title

1a-iii) Primary condition or target group in the title

"for Patients with Non-specific Low Back Pain"

ABSTRACT

1b-i) Key features/functionalities/components of the intervention and comparator in the METHODS section of the ABSTRACT

"a user-centered telerehabilitation program, comprised of a smartphone application and integrated sensors,"

1b-ii) Level of human involvement in the METHODS section of the ABSTRACT

1b-iii) Open vs. closed, web-based (self-assessment) vs. face-to-face assessments in the METHODS section of the ABSTRACT

"Patients in TBEG completed digital assessment questionnairesvia the application at weeks 0, 2, 4, and 8."

1b-iv) RESULTS section in abstract must contain use data

"Patients in the OBEG have a completion rate of 96.30% (26/27) at 2 weeks, 92.59% (25/27) at 4 weeks, and 81.48% (22/27) at 8 weeks. Patients in the TBEG have a completion rate of 100% (27/27) at 2 weeks, 96.30% (26/27) at 4 weeks, and 88.89% (24/27) at 8 weeks"

1b-v) CONCLUSIONS/DISCUSSION in abstract for negative trials

INTRODUCTION

2a-i) Problem and the type of system/solution

“ As a result, the traditional clinic-based exercise model encounters difficulties in addressing the diverse needs of this patient population. Given these circumstances, the incorporation of home-based exercises into a telerehabilitation program emerges as a promising and effective strategy to address the previously mentioned challenges associated with managing NLBP.In developing nations such as China, the widespread adoption of telerehabilitation is important because of the large patient population and the lack of physical therapy services.“

2a-ii) Scientific background, rationale: What is known about the (type of) system

“ in the United Kingdom, Fatoye F et al integrated telerehabilitation with the McKenzie exercise approach. Remarkably, the telerehabilitation group achieved therapeutic outcomes equivalent to outpatient rehabilitation. It also demonstrated lower average medical cost per patient compared to the outpatient group”

Does your paper address CONSORT subitem 2b?

"The research team used the Healbone Intelligent Rehabilitation System (HIRS), comprised of a smartphone application and integrated sensors. The primary objective is to evaluate the efficacy of this intervention. The program guides and monitors NLBP patients as they engage in a structured home-based exercise regimen. This study measures the program's impact on both the physical and psychological dimensions of NLBP management."

METHODS

3a) CONSORT: Description of trial design (such as parallel, factorial) including allocation ratio

"This study was a single-center, 2-arm, parallel-group RCT (participant-blinded) with 1:1 randomization conducted in Peking Union Medical College Hospital, Beijing,China."

3b) CONSORT: Important changes to methods after trial commencement (such as eligibility criteria), with reasons

No significant changes in methodology have occurred since the start of this study

3b-i) Bug fixes, Downtimes, Content Changes

4a) CONSORT: Eligibility criteria for participants

“Inclusion criteria:
Aged between 18 and 60 years;
Numeric Pain Rating Scale (NPRS) equal to or greater than 3 points;
Oswestry Disability Index(ODI) equal to or greater than 15 points;
Ongoing pain for at least 3 months;
Able to use smartphone and complete the exercise protocol independently;
Those who could sign the informed consent independently;
Exclusion criteria:
Patients with Spinal deformity, spinal structure slip, spinal fracture history, spinal tumor;
Diagnosed rheumatoid arthritis and ankylosing spondylitis;
Patients with herniated disc;
Pregnancy;
Patients who receive other treatments before the experiments, including non-steroid anti-inflammatory drugs or plasters, physical agents therapy, and acupuncture.“

4a-i) Computer / Internet literacy

“Able to use smartphone and complete the exercise protocol independently;”

4a-ii) Open vs. closed, web-based vs. face-to-face assessments:

“In this study, all patients with NLBP were recruited from Peking Union Medical College Hospital.”

4a-iii) Information giving during recruitment

"The researchers were required to explain the purposes, procedures, and possible risks of the trial in detail to the patients before inclusion. Written informed consents were obtained from all patients."

4b) CONSORT: Settings and locations where the data were collected

"patients were required to complete digital assessment questionnaires via the application at weeks 0, 2, 4, and 8."

4b-i) Report if outcomes were (self-)assessed through online questionnaires

"patients were required to complete digital assessment questionnaires via the application at weeks 0, 2, 4, and 8.."

4b-ii) Report how institutional affiliations are displayed

5) CONSORT: Describe the interventions for each group with sufficient details to allow replication, including how and when they were actually administered

5-i) Mention names, credential, affiliations of the developers, sponsors, and owners

5-ii) Describe the history/development process

5-iii) Revisions and updating

5-iv) Quality assurance methods

**5-v) Ensure replicability by publishing the source code, and/or providing screenshots/screen-capture video, and/or providing flowcharts of the algorithms used**		
**5-vi) Digital preservation**		
"the transmitter portal encrypts and transmits the data collected, ensuring the overall system's integrity."		
**5-vii) Access**		
" And simutaneously, the HJHS was free to all participants in this study. Initially, an application was installed on the smartphones of the TBEG patients. Subsequently, the PTs delivered detailed instructions on the exercises during the first session, educating the patients on the proper usage of the application and sensors for home exercises."		
**5-viii) Mode of delivery, features/functionalities/components of the intervention and comparator, and the theoretical framework**		
"The HIRS was designed based on an user-centered theory, to provide patients with a platform for self-management interventions. Initially, an application was installed on the smartphones of the TBEG patients. Subsequently, the PTs delivered detailed instructions on the exercises during the first session, educating the patients on the proper usage of the application and sensors for home exercises."		
**5-ix) Describe use parameters**		
"During the 8-week period, all TBEG patients were required to complete a 30-minute exercise routines every 2 days, 3 times per week. PTs regularly communicated with their patients through the application to address any quesitons or concerns. Additionally, the application also allowed the PTs to send reminders to patients who did not complete their exercises on time."		
**5-x) Clarify the level of human involvement**		
**5-xi) Report any prompts/reminders used**		
"Additionally, the application also allowed the PTs to send reminders to patients who did not complete their exercises on time."		
**5-xii) Describe any co-interventions (incl. training/support)**		
"At the initiation of this trial, two physical therapists (PT) were trained in three 40-minute sessions."		
**6a) CONSORT: Completely defined pre-specified primary and secondary outcome measures, including how and when they were assessed**		
"These measures include the NPRS for pain evaluation, the SF-36 for quality of life assessment, and the FABQ to gauge fear-avoidance beliefs related to work and physical activity. The reliability and validity of the Chinese version of the SF-36 and the FABQ have been confirmed. The collection of the primary and secondary outcome measures occurred at weeks 0,2,4, and 8"		
**6a-i) Online questionnaires: describe if they were validated for online use and apply CHERRIES items to describe how the questionnaires were designed/deployed**		
**6a-ii) Describe whether and how "use" (including intensity of use/dosage) was defined/measured/monitored**		
**6a-iii) Describe whether, how, and when qualitative feedback from participants was obtained**		
**6b) CONSORT: Any changes to trial outcomes after the trial commenced, with reasons**		
"patients were required to complete digital assessment questionnaires via the application at weeks 0, 2, 4, and 8."		
**7a) CONSORT: How sample size was determined**		
**7a-i) Describe whether and how expected attrition was taken into account when calculating the sample size**		
"The sample size was calculated using PASS 11. Based on previous clinical studies[17,25], the mean difference in ODI between the TBEG and the OBEG was 5, the standard deviation was estimated to be 6 for both groups, and the non-inferiority margin for ODI was 10. A sample size of 38 was required based on a bilateral alpha 0.05 and beta=0.2, and 54 for a 30% dropout rate."		
**7b) CONSORT: When applicable, explanation of any interim analyses and stopping guidelines**		
"These measures include the NPRS for pain evaluation, the SF-36 for quality of life assessment, and the FABQ to gauge fear-avoidance beliefs related to work and physical activity. The reliability and validity of the Chinese version of the SF-36 and the FABQ have been confirmed. The collection of the primary and secondary outcome measures occurred at weeks 0,2,4, and 8"		
**8a) CONSORT: Method used to generate the random allocation sequence**		
"Subjects were assigned in a randomized manner, with equal distribution, to either the TBEG or the OBEG through an online platform (https://www.random.org/). "		
**8b) CONSORT: Type of randomisation; details of any restriction (such as blocking and block size)**		
"The allocation sequence was prepared by a researcher with no involvement in the study using a blocked randomization model. "		
**9) CONSORT: Mechanism used to implement the random allocation sequence (such as sequentially numbered containers), describing any steps taken to conceal the sequence until interventions were assigned**		
Allocation concealment was ensured using sequentially numbered opaque and sealed envelopes.		
**10) CONSORT: Who generated the random allocation sequence, who enrolled participants, and who assigned participants to interventions**		
"Houqiang Zhang was involved in random number generation, Lixia Chen was involved in patient recruitment, and Weihong Shi and Yuhang Zhang were involved in the intervention"		
**11a) CONSORT: Blinding - If done, who was blinded after assignment to interventions (for example, participants, care providers, those assessing outcomes) and how**		
**11a-i) Specify who was blinded, and who wasn't**		
"The allocation sequence was prepared by a researcher with no involvement in the study using a blocked randomization model.The Statistical Analysis was conducted by a researcher with no involvement in the study. "		
**11a-ii) Discuss e.g., whether participants knew which intervention was the "intervention of interest" and which one was the "comparator"**		
**11b) CONSORT: If relevant, description of the similarity of interventions**		
"The exercise plan for patients with NLBP in both TBEG and OBEG was identical. "		
**12a) CONSORT: Statistical methods used to compare groups for primary and secondary outcomes**		
"The analysis was conducted using the intent-to-treat approach, participants were analyzed according to the original group assignment. The baseline data for those who failed to follow up were included. All the data in this study was analyzed using IBM SPSS 23.0. Demographic data are presented as means (standard deviation) and numbers (percentage). Descriptive statistics, independent sample t-tests, and Chi-squared tests were used to analyze participant characteristics. The normality of distribution for all data was tested by an independent sample t-test. The results of this study are presented as mean, standard deviation (SD), and 95% confidence interval (CI)."		
**12a-i) Imputation techniques to deal with attrition / missing values**		
"The analysis was conducted using the intent-to-treat approach, participants were analyzed according to the original group assignment."		
**12b) CONSORT: Methods for additional analyses, such as subgroup analyses and adjusted analyses**		
No adjusted analysis was performed in this study, and the dropping rate was within the 30% allowed.		
RESULTS		
**13a) CONSORT: For each group, the numbers of participants who were randomly assigned, received intended treatment, and were analysed for the primary outcome**		
"At the initiation of this trial, two physical therapists (PT) were trained in three 40-minute sessions."		
**13b) CONSORT: For each group, losses and exclusions after randomisation, together with reasons**		
"Figure 2. Participant flowchart"		
**13b-i) Attrition diagram**		
"Figure 2. Participant flowchart"		
**14a) CONSORT: Dates defining the periods of recruitment and follow-up**		
"Between March 9th, 2023 and November 1st, 2023, 128 patients were considered for eligibility. "		
**14a-i) Indicate if critical "secular events" fell into the study period**		
**14b) CONSORT: Why the trial ended or was stopped (early)**		

The study did not end early		
**15) CONSORT: A table showing baseline demographic and clinical characteristics for each group**		
"The population was randomly allocated into two groups: TBEG (n = 27) and OBEG (n = 27), as illistrated in Figure 2."		
**15-i) Report demographics associated with digital divide issues**		
"Table 2 Demographics and baseline characteristics of all participants."		
**16a) CONSORT: For each group, number of participants (denominator) included in each analysis and whether the analysis was by original assigned groups**		
**16-i) Report multiple "denominators" and provide definitions**		
"All patients completed the baseline assessment at week 0 and were asked to complete assessments at weeks 2, 4, and 8. Patients in the OBEG have a completion rate of 96.30% (26/27) at 2 weeks, 92.59% (25/27) at 4 weeks, and 81.48% (22/27) at 8 weeks. Patients in the TBEG have a completion rate of 100% (27/27) at 2 weeks, 96.30% (26/27) at 4 weeks, and 88.89% (24/27) at 8 weeks, as illustrated in Figure 2."		
**16-ii) Primary analysis should be intent-to-treat**		
**17a) CONSORT: For each primary and secondary outcome, results for each group, and the estimated effect size and its precision (such as 95% confidence interval)**		
"Table 3 Primary and secondary outcomes for the OBEG and the TBEG."		
**17a-i) Presentation of process outcomes such as metrics of use and intensity of use**		
**17b) CONSORT: For binary outcomes, presentation of both absolute and relative effect sizes is recommended**		
This study does not involve binary outcomes		
**18) CONSORT: Results of any other analyses performed, including subgroup analyses and adjusted analyses, distinguishing pre-specified from exploratory**		
This Results of any other analyses performed.		
**18-i) Subgroup analysis of comparing only users**		
**19) CONSORT: All important harms or unintended effects in each group**		
There is no any harm or unintended effect in each group of this study		
**19-i) Include privacy breaches, technical problems**		
**19-ii) Include qualitative feedback from participants or observations from staff/researchers**		
DISCUSSION		
**20) CONSORT: Trial limitations, addressing sources of potential bias, imprecision, multiplicity of analyses**		
**20-i) Typical limitations in ehealth trials**		
"Participants in ehealth trials are rarely blinded, which might increase risk for a Type I error. The number of participants is relatively small and follow-up period also short . To overcome these challenges, the research team planned for a subsequent multi-center randomized controlled trial. This future study will involve a greater number of participants who meet the same criteria as in the initial study."		
**21) CONSORT: Generalisability (external validity, applicability) of the trial findings**		
**21-i) Generalizability to other populations**		
**21-ii) Discuss if there were elements in the RCT that would be different in a routine application setting**		
**22) CONSORT: Interpretation consistent with results, balancing benefits and harms, and considering other relevant evidence**		
**22-i) Restate study questions and summarize the answers suggested by the data, starting with primary outcomes and process outcomes (use)**		
"This study is designed to determine the efficacy of the treatment between the TBEG and the OBEG. After 8-week intervention, the completion rate was 88.89% in the TBEG and 81.48% in the OBEG. Although the completion rate in two groups is similar, Patients in the TBEG have more fexibility on the exercise shedule and they always complete exercise plan timely. Results show that there was no statistical difference in pain relief and improvement of pain-related physical dysfunction and quality of life between the TBEG and OBEG after 8-week intervention. As compared with previous studies[29,30], this study also demonstrates the efficacy of telerehabilitation for patients with NLBP in the improvement of pain intensity, physical disability and life of quality. "		
**22-ii) Highlight unanswered new questions, suggest future research**		
Other information		
**23) CONSORT: Registration number and name of trial registry**		
Chinese Clinical Trial Registry: ChiCTR2300068984 ;<br>(https://www.chictr.org.cn/showproj.html?proj=189852)		
**24) CONSORT: Where the full trial protocol can be accessed, if available**		
Chinese Clinical Trial Registry: ChiCTR2300068984 ;<br>(https://www.chictr.org.cn/showproj.html?proj=189852)		
**25) CONSORT: Sources of funding and other support (such as supply of drugs), role of funders**		
National High-Level Hospital Clinical Research Funding (2022-PUMCH-B-053)		
**X26-i) Comment on ethics committee approval**		
**x26-ii) Outline informed consent procedures**		
**X26-iii) Safety and security procedures**		
**X27-i) State the relation of the study team towards the system being evaluated**		
